# Hepatitis C Virus Life Cycle and Lipid Metabolism

**DOI:** 10.3390/biology3040892

**Published:** 2014-12-15

**Authors:** Costin-Ioan Popescu, Laura Riva, Ovidiu Vlaicu, Rayan Farhat, Yves Rouillé, Jean Dubuisson

**Affiliations:** 1 Institute of Biochemistry of the Romanian Academy, Splaiul Independentei 296, 060031 Bucharest 17, Romania; E-Mails: pop@biochim.ro (C.-I.P.); vlaicu.ovidiu@yahoo.com (O.V.); 2 Center for Infection & Immunity of Lille (CIIL), Inserm U1019, CNRS UMR8204, Institut Pasteur de Lille and Université de Lille, F-59000 Lille, France; E-Mails: laura.riva@ibl.cnrs.fr (L.R.); rayan.farhat@ibl.cnrs.fr (R.F.); yves.rouille@ibl.cnrs.fr (Y.R.)

**Keywords:** Hepatitis C virus, viral life cycle, lipid metabolism, virus-host interactions

## Abstract

Hepatitis C Virus (HCV) infects over 150 million people worldwide. In most cases HCV infection becomes chronic, causing liver disease ranging from fibrosis to cirrhosis and hepatocellular carcinoma. HCV affects the cholesterol homeostasis and at the molecular level, every step of the virus life cycle is intimately connected to lipid metabolism. In this review, we present an update on the lipids and apolipoproteins that are involved in the HCV infectious cycle steps: entry, replication and assembly. Moreover, the result of the assembly process is a lipoviroparticle, which represents a peculiarity of hepatitis C virion. This review illustrates an example of an intricate virus-host interaction governed by lipid metabolism.

## 1. Introduction

Hepatitis C Virus (HCV) affects over 150 million people worldwide. The majority of infections evolve to chronicity and liver disease starting from steatosis and fibrosis to cirrhosis and hepatocellular carcinoma [1]. Until recently, the standard for therapy has been represented by pegylated interferon alpha plus ribavirin. The treatment had significant side effects and variable efficacy depending on the viral genotype. In the last two years, HCV therapy has been profoundly improved with the approval of direct acting antivirals in the clinical practice (reviewed in [2]). The new gold standard for treatment has a better sustained virological response rate with significant reduction of the treatment period and less side effects. Despite significant advances in HCV therapy, the drug resistance and genotype specific efficacy are still issues to be considered.

HCV infection affects lipid metabolism and cholesterol homeostasis in particular. The association of HCV with lipid metabolism has long been noticed in clinical practice. Liver biopsies of infected patients present an increase of neutral lipids in cytosolic lipid droplets [3]. Non-A and non-B hepatitis has been associated with liver steatosis, frequent hypobetalipoproteinemia and reduced blood levels of cholesterol (reviewed in [4]). It is worth noting that patients infected by genotype 3 viruses are more prone to severe steatosis, suggesting that specific viral sequences are responsible for lipid accumulation in the liver (reviewed in [5]). Although several mechanisms have been proposed to account for the viral steatosis, no experimental model clearly recapitulates the phenotype observed in humans. HCV treatment by interferon alpha and ribavirin restores the cholesterol and the lipoproteins levels in patient sera [6]. At the same time, initial virus purification from infected patients sera revealed the low density of the virions and their association with apolipoproteins [7].

**Figure 1:**
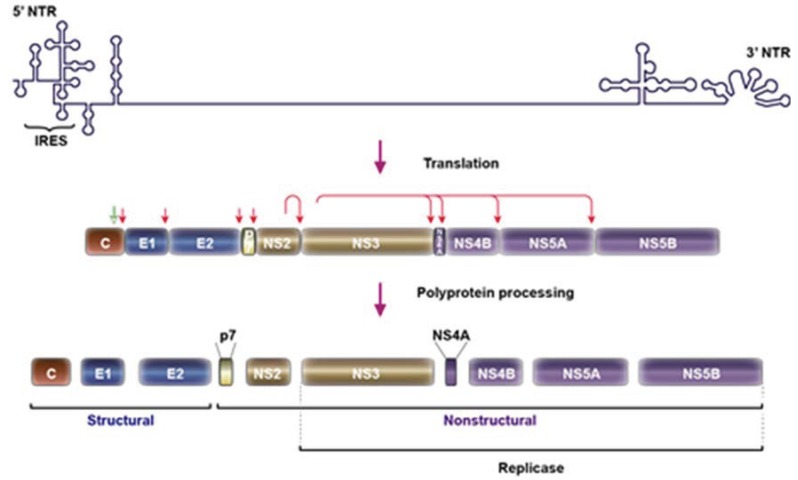
Genomic organization of HCV and protein synthesis. The HCV genome contains a single open reading frame flanked by 5ꞌ and 3ꞌ non-coding regions. The 5ꞌ NTR contains an internal ribosome entry site (IRES). After its synthesis, the HCV polyprotein is cleaved by host signal peptidase (red vertical arrows) and by viral encoded proteases (NS2 and NS3/4A) as indicated by corresponding arrows. An additional cleavage removing the carboxy-terminal region of the core protein is mediated by cellular signal peptide peptidase (green vertical arrow). The functions of the individual proteins are indicated in the text.

HCV is an enveloped positive-stranded RNA virus that belongs to the *Flaviviridae* family. The viral genome is translated into a polypeptide, which is sequentially processed into ten mature proteins (Figure 1). The structural proteins core and the envelope proteins (E1 and E2) lie at the N-terminus of the polyprotein. The non-structural proteins NS3, NS4A, NS4B, NS5A and NS5B are located at the C-terminus. Between the structural and the non-structural proteins are two proteins most likely non-structural (p7 and NS2), which are dispensable for replication, but essential for assembly [8].

Core protein associates with the viral RNA to form the nucleocapsid. E1 and E2 envelope glycoproteins form a heterodimer, which is most likely the functional unit of the viral envelope. The p7 polypeptide is a small hydrophobic protein, which forms an ion channel, and it is involved in viral assembly and secretion. NS2 is a multifunctional protein essential for both assembly and replication by its function as an autocatalytic cysteine protease. The N-terminal domain of NS3 is the second viral protease that processes the viral polypeptide towards the C-terminus, whereas the C-terminal domain of NS3 has a helicase function. NS4A is a small hydrophobic protein that serves as a cofactor for NS3 serine protease. NS4B protein induces the rearrangement of the intracellular membranes assuring the framework for viral replication. NS5A is a multifunctional protein involved in replication and assembly. NS5B is the viral RNA-dependent RNA polymerase that forms a replication complex together with NS3, NS4A, NS4B and NS5A [9].

Over twenty-five years of research has revealed the molecular mechanisms of the association between HCV and lipid metabolism. The emergence of an infectious system able to sustain robust amplification of HCV in cell culture (HCVcc) boosted our understanding of the role of lipids in each step of the viral life cycle [10,11,12]. Lipid metabolism is deeply involved in the molecular mechanisms of the HCV infectious cycle. While HCV is a lipoviroparticle that uses lipid-related factors for entry, viral replication is associated with profound changes of intracellular membrane architecture and viral assembly and secretion take place in the microenvironment of the endoplasmic reticulum (ER) and lipid droplets (LD) overlapping with the very-low density lipoprotein (VLDL) secretion pathway. The aim of this review is to present an update on HCV-lipid metabolism interactions.

## 2. HCV Particle

Before the development of a cell culture system for HCV (HCVcc), scientists relied on virus isolated from chronically infected patients or experimentally inoculated chimpanzees for the biophysical and ultrastructural characterization of the virion. Such studies rapidly revealed that HCV particles exhibit a surprisingly low density and are distributed over a wide range of densities, ranging from 1.03 g/cm^3^ to 1.20 g/cm^3^ when analyzed in sucrose gradients [13], and the lower density fractions are the most infectious [14]. Surprisingly, dietary triglyceride alters the density and dynamics of HCV in plasma of chronically infected patients [15]. These unusual biophysical features are due to the presence of lipoproteins associated with HCV particles [7,16,17], and they have been confirmed in the HCVcc system [18]. Indeed, apolipoproteins (apo) such as apoE, apoB, apoA1 and several apoC proteins can be found in association with HCV particles [19,20,21,22,23]. Furthermore, the lipid composition of HCV virions indicates that more than half of the total HCV lipids are composed of cholesteryl esters, which resembles the lipid content of VLDL and low-density lipoproteins (LDL) [24]. Due to their association with lipoproteins, HCV particles were named lipoviroparticles (LVP) [7]. Interestingly, LVP are enriched in apoE as compared to VLDL. Indeed, purified HCVcc particles bear approximately 300 molecules of apoE [24], whereas VLDL contains only five to seven apoE molecules [25]. However, the average density of HCV particles isolated *in vivo* is lower compared to HCVcc produced *in vitro* [18]. This is likely due to some defect in VLDL biogenesis in Huh-7 [26], the hepatoma cell line generally used to produce HCV in cell culture. These cells are indeed deficient in producing mature VLDL due to poor efficiency of apoB100 lipidation.

HCV particles are 50–80 nm in diameter [19] and contain classical viral components such as a single-stranded RNA genome, core and the envelope glycoproteins, E1 and E2 [27]. HCV genome interacts with the core protein to form the nucleocapsid that is surrounded by a lipid membrane, called the viral envelope, in which are anchored the envelope glycoproteins. The exact nature of the interactions involved between HCV virion components and the lipoprotein remains poorly understood. One possibility could be that lipoproteins peripherally associate with canonical viral particles via interaction between apolipoproteins and HCV envelope lipids or proteins [28]. Alternatively, HCV virion could be a hybrid particle composed of a virion moiety and a lipoprotein component [29]. This latter hypothesis is reinforced by the observation that recombinant HCV envelope glycoproteins, expressed in the absence of other viral components, can be secreted along with triglyceride rich lipoproteins by differentiated intestinal Caco-2 cells [30]. These recombinant envelope glycoproteins were shown to be associated with lipoproteins containing apoB. More recently, it has also been reported that HCV envelope glycoproteins directly interact with apoE and apoB during HCV morphogenesis [31,32]. Whatever the mode of association, HCV interaction with lipoproteins could contribute to the shielding of the viral envelope glycoproteins from the host antibody neutralizing response and could explain the poor detection or availability of HCV glycoproteins at the virion surface [19,24,33].

## 3. HCV Entry

Due to difficulties in propagating the virus in cell culture, functional studies on HCV entry were only initiated with the development of retroviral pseudotypes harboring HCV envelope glycoproteins, which are usually called HCV pseudoparticles (HCVpp) [34,35,36]. HCVpp entry depends on the functions of HCV envelope glycoproteins present at their surface. However, HCVpp cannot entirely mimic HCV entry since, in contrast to HCV virions, they do not seem to be associated with VLDL [34]. The development of the HCVcc system has therefore been a major progress to better understand the role of lipid metabolism in HCV entry. Importantly, the peculiar hybrid composition of HCV particle provides an opportunity for the virus to interact with lipoprotein receptors during the early steps of viral entry.

**Figure 2:**
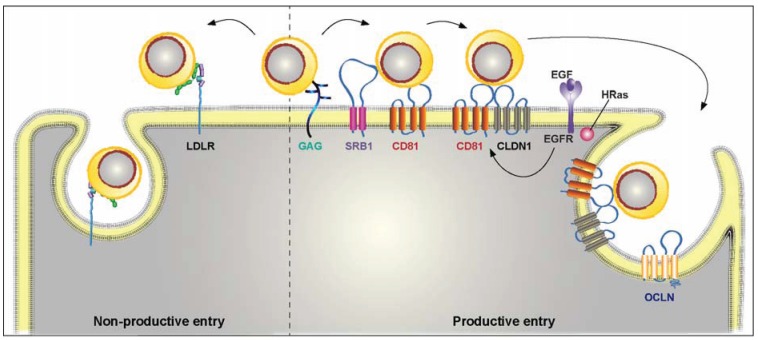
Cellular entry of HCV particles. HCV virion is associated with lipoproteins to form a complex particle that has been called lipoviroparticle (LVP). It initiates its life cycle by binding to glycosaminoglycans (GAGs). Then the virus can follow either a productive or a non-productive pathway. In the non-productive pathway, the lipoprotein component of the viral particle interacts with the LDL receptor (LDL-R) and the virion is rapidly internalized and potentially sent to a degradation pathway. The productive pathway is a complex multistep process involving a series of specific cellular entry factors, which include SRB1, CD81, tight-junction proteins, CLDN1 and OCLN, as well as other cellular factors not represented in this figure. After binding to several components of the host cell, HCV particle is internalized by clathrin-mediated endocytosis and fusion takes place in early endosomes.

HCV entry is initiated by the binding of virions to attachment factors present at the surface of hepatocytes. As for many viruses, initial attachment of HCV particles to hepatocytes is mediated by virion binding to heparan sulfate proteoglycans [37] (Figure 2). Recently, it has been shown that HCV virion mainly uses syndecan-1 heparan sulfate proteoglycan to initiate entry into human hepatocytes [38]. However, another study suggests that HCV particle might preferentially use syndecan-4 [39]. Therefore, one cannot exclude that several proteoglycans can be used for the initial binding of HCV. Before the development of a cell culture system for HCV, it was first proposed that the envelope glycoproteins are the viral components involved in binding to heparan sulfate as these viral glycoproteins can bind to heparin [40]. However, apoE, which is present at the surface of HCV virion [41], is also able to interact with heparan sulfate and more recent studies suggest that this apolipoprotein could be the viral component involved in this interaction [42].

HCV particle tethering to heparan sulfate moieties likely helps the virus to stay localized at the surface of the hepatocyte while sampling the adjacent molecules to find more specific entry factors. The association of HCV particle with VLDL provides the opportunity for this virus to interact with lipoprotein receptors, and it was first believed that the LDL receptor could be used by HCV to enter the hepatocyte [43,44,45]. However, more recent studies suggest that HCV interaction with the LDL receptor would rather involve a non-productive entry pathway that can potentially lead to viral particle degradation [46]. Interestingly, lipoprotein lipase has been shown to modulate HCV entry [47]. This enzyme increases HCV attachment to host cells, but its catalytic activity reduces HCV infectivity by changing the lipid and apolipoprotein composition of the viral particle [46,47,48].

Another lipoprotein receptor, SRB1, is also involved in HCV entry. SRB1 is a rather promiscuous receptor that binds ligands such as HDL, LDL, VLDL remnants and oxidized and acetylated LDL, to name a few [49]. In contrast to the LDL receptor, SRB1 plays a functional role in the productive entry pathway of HCV. In this case, a direct interaction between HCV envelope glycoprotein E2 and SRB1 lipoprotein receptor has first been shown in which hypervariable region 1 (HVR1) of E2 plays an essential role [50]. However, SRB1 also seems to contribute to virus attachment through interaction with virion-associated lipoproteins [33,51]. Furthermore, the role of SRB1 in HCV entry could be even more complex than previously thought since this receptor also mediates a post-binding event important for productive viral entry [52,53]. One possibility to explain these multiple functions is that, after interaction with heparan sulfate, the lipoprotein associated with the virion could interact with SRB1 leading to the binding of HVR1 of E2 to SRB1. Next, SRB1, through its lipid transfer activity, could modify the lipid composition of the lipoprotein moiety of the virion and/or modify the local lipid composition of the plasma membrane, which would favor the interaction between HCV particle and other cellular entry factors. However additional experiments are needed to better understand the complex implication of SRB1 in HCV entry. It is worth noting that HCV entry can be modulated by SRB1 ligands. Indeed, HDL has been shown to enhance HCV entry in a process that depends on the lipid transfer function of SRB1 and the presence of apoC1 [54,55,56]. In contrast, oxidized LDLs inhibit HCV entry [57].

Since HCV particles can potentially bind both LDL receptor and SRB1, one can wonder what would preferentially orient HCV entry towards a productive infection by interacting with SRB1 instead of the LDL receptor. One possibility is that the particular lipid and apolipoprotein composition of the virion circulating in the bloodstream endows the virus with a higher affinity for SRB1. Furthermore, since HCV glycoprotein E2 also interacts with SRB1, it is possible that HCV binding to SRB1 is stabilized by this interaction before transferring the virion to the next receptor. However, one cannot exclude that virion remodeling by lipoprotein lipase in the bloodstream could also lead to some binding to the LDL receptor.

After binding to SRB1, the HCV particle seems to interact with the tetraspanin molecule CD81 [58]. Importantly, it is currently believed that SRB1 interaction with HVR1 could unmask the CD81 binding site of E2, as suggested by the reduced dependence on SRB1 of HVR1-deleted mutant viruses [59,60]. Among HCV entry factors, CD81 is undoubtedly a central player in the early steps of the HCV life cycle [61]. This tetraspanin is highly dynamic at the cell surface and it is enriched in areas of the membrane that form stable platforms, which are in permanent exchange with the rest of the membrane [62]. Importantly, the balance of these dynamic exchanges in the cell membrane is essential for HCV entry. Indeed, CD81 molecules that freely diffuse and are therefore not engaged in static microdomains are preferentially used by HCV during virus entry [63,64]. Importantly, alteration of the lipid composition of the plasma membrane can modulate CD81-dependent HCV entry into host cells. Indeed, depletion of cholesterol from the plasma membrane or altering the sphingomyelin/ceramide ratio of the plasma membrane affects HCV entry by reducing the cell surface expression of CD81 [65,66].

In addition to CD81, two tight-junction proteins, Claudin-1 (CLDN1) and Occludin (OCLN), have also been shown to be essential for HCV entry [67,68]. Importantly, CLDN1 forms a co-receptor complex with CD81 [69,70], which is involved in downstream events of HCV entry [71]. This CD81-CLDN1 association appears to be regulated by multiple signaling pathways (for review in [72]). Notably, the epidermal growth factor receptor (EGFR) promotes CD81-CLDN1 complex formation by inducing CD81 diffusion through HRas activation and facilitates CD81-CLDN1 co-internalization with HCV particles [73,74]. The role of OCLN in the HCV life cycle remains poorly understood. It seems to play a role at a late entry step [75]. It has to be noted that OCLN depletion impairing HCV entry does not perturb CLDN1 expression or localization, suggesting that both entry factors function separately during HCV infection [76,77].

More recently, another protein involved in lipid metabolism was also identified as an additional entry factor. Indeed, the cholesterol transporter Niemann-Pick C1-like 1 (NPC1L1) was shown to play a role in HCV entry [78]. NPC1L1 is a cholesterol transport protein principally located on the apical surface of hepatocytes, facing the bile canaliculi, and it plays a role in regulating hepatic cholesterol levels by reabsorbing biliary cholesterol secreted in the bile. Since this receptor is located at the apical membrane of hepatocytes and HCV entry is supposed to occur at the basolateral membrane, the role of NPC1L1 may occur indirectly via cholesterol regulation.

After successive binding to several entry factors at the hepatocyte surface, the HCV particle is endocytosed by a clathrin-dependent process [79], in association with CD81-CLDN1 complexes [71,80]. However, alternative entry routes have also been reported [81]. In Huh-7 cells, the HCV virion is transported to Rab5a positive early endosomes along actin stress fibers, where fusion between the viral envelope and a cellular membrane has been reported to take place [80]. Interestingly, E2 interaction with CD81 seems to prime HCV envelope proteins for low pH-dependent fusion [82]. However, recent structural data of E2 glycoprotein [83,84] suggest that, contrary to a previous hypothesis [85], this protein is not a fusion protein. These new observations strongly suggest that E1 should be the fusion protein or, at least, a fusion partner of an E1E2 fusion complex formed upon conformational rearrangements [86].

## 4. HCV Replication

### 4.1. HCV-Induced Membrane Rearrangements

**Figure 3:**
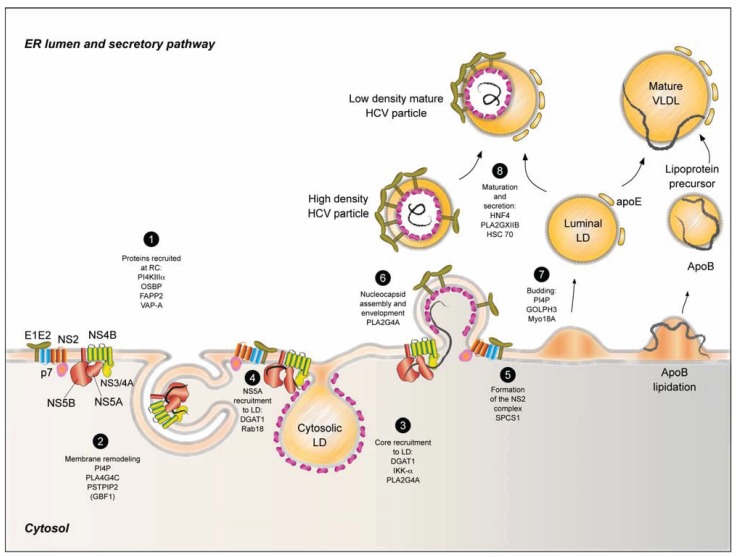
Model of viral replication and assembly of the infectious viral particle. Following viral protein processing, the mature proteins assemble in complexes. The NS2 complex consists of the envelope proteins E1 and E2, p7 viroporin and NS2 protease. The replication complex gather NS3 protease and its co-factor NS4A, NS4B, NS5A and NS5B, which are associated with modified membranes forming the “membranous web”. The cellular proteins PI4KIIIalpha, OSBP, FAPP2 and VAP-A are also recruited to the replication complexes, regulating membrane lipid exchange (**1**). NS4B protein induces the reorganization of intracellular membranes in special partially closed double membrane structures where replication occurs. The host proteins PLA2G4C and PSTPIP2 influence this membrane remodeling. Furthermore, GBF1 is involved in an early step of viral replication and it indirectly affects membrane remodeling (**2**). In the early stage of assembly core associates with LD following the recruitment of the replication complex through core-NS5A interaction. Several endogenous factors are involved in core and NS5A recruitment to LD and DGAT1 plays a central role in this relocalization (**3** and **4**). NS2 complexes (**5**) may interact with replication complexes accumulating in dotted structures in the proximity of LD. The presence of the three viral modules triggers the synchronous nucleocapsid assembly and envelopment potentiated by endogenous factors like PLA2G4A (**6**). Following the host-mediated budding (**7**), the immature viral particle fuses or attaches to a luminal lipid droplet through apoE-E1E2 interaction. The LVP takes a similar secretion and maturation route as VLDL, influenced by the cellular proteins HNF4, PLA2GXIIB and HSC70 (**8**).

Not all the non-structural viral proteins are required for replication of the viral genome. Studies using replicons, which are minimal replication units, have indicated that proteins NS3-4A, NS4B, NS5A and NS5B, together with both untranslated regions (UTR), are necessary and sufficient for the replication [87]. HCV replication occurs in association with rearranged intracellular membranes, which have been named “membranous web” (Figure 3). The membranous web was initially described in U-2 OS cells inducibly expressing the HCV polyprotein [88], indicating that its formation does not depend on the active replication of viral RNA, but only on the expression of non-structural proteins. In this model, it was shown to be composed of small vesicles embedded in a membrane matrix. Similar membrane alterations were later observed in Huh-7 cells harboring a subgenomic replicon of genotype 1b [89] and in JFH1-infected Huh-7 cells [90]. In replicon-containing cells, the membranous web was reported to contain the non-structural proteins NS3/4A, NS4B, NS5A and NS5B, and the genomic RNA. Moreover, newly synthesized viral RNA was also detected in the membranous web, indicating that it is a site of viral RNA synthesis [89]. Later on, replication-induced membrane rearrangements were shown to be more complex than initially thought. In addition to the multi-vesicular structures initially observed, the use of a GFP-tagged replicon indicated that the membranous web also comprises small membrane structures highly mobile and scattered throughout the cytoplasm of the cell [91]. Using highly permissive Huh-7.5 cells replicating a subgenomic replicon of the JFH1 strain, the membrane alterations were shown to include numerous double membrane vesicles [92] that had not been observed before with replicons of genotype 1b. These double membrane vesicles, together with single membrane vesicles were also observed in JFH1-infected Huh-7.5 or Lunet cells [93,94]. Double membrane vesicles were also observed in cells replicating enteroviruses [95,96] or coronaviruses [97,98]. However, they were not observed in cells infected with other *Flaviviridae* family members, such as flaviviruses, which replicate their genome in invaginations of the ER membrane [99,100], or pestiviruses, for which replication does not apparently correlate with any specific membrane rearrangements [101]. The absence of membrane-associated ribosomes suggests that the different types of membrane alteration observed in HCV-infected cells are not involved in translation of the genome. Their exact function during the replication step of the HCV life cycle is not yet clearly defined.

The formation and the activity of the membranous web are still poorly understood. NS4B and NS5A play a major role in the induction of membrane rearrangements. The expression of NS4B protein alone induces membrane alterations similar to membranous webs of infected cells [88,89] and the expression of NS5A induces the formation of double membrane vesicles [94]. Both morphological and biochemical data led to the proposal that HCV replication complexes are derived from the ER membrane [88,89,91,94,102]. However, several endosomal markers were also reported as being colocalized with replication complexes and/or functionally involved in RNA replication [103,104,105]. Moreover, it has also been shown that RNA replication occurs in detergent-resistant membranes [106]. This indicates that the membranes of HCV replication complexes are enriched in cholesterol and sphingolipids, two lipids underrepresented in the ER membrane. Therefore, these lipids have to be transported to the replication complexes, or the replication complexes form in sub-domains of the ER membrane locally enriched in cholesterol and sphingolipids. All these data suggest that the membranes of the HCV replicase could be derived from the ER membrane and biochemically modified to create a particular lipid environment required for the replicative activity of the complexes. The formation of HCV replication complexes from the membrane of the ER would therefore involve coordinated biochemical and morphological changes.

### 4.2. Role of Phosphatidylinositol-4 Phosphate in HCV Replication

One major host factor implicated in HCV RNA replication, which has been found in several simultaneous siRNA screens, is the phosphatidylinositol-4 kinase-IIIα (PI4KIIIα, also known as PI4KA) [103,105,107,108,109,110]. PI4KIIIα interacts with, and is activated by NS5A during HCV replication [111,112,113]. In infected cells, PI4KIIIα is recruited to the replication complexes away from its normal localization at the ER and plasma membranes. Small molecule inhibitors of PI4KIIIα inhibit HCV replication [114,115]. PI4KIIIα depletion leads to morphologically aberrant NS5A-positive structures in cells expressing the HCV polyprotein [94,105,112], and this phenotype is mimicked by both NS5A and PI4KIIIα inhibitors [113,115,116,117,118,119]. PI4KIIIα activation leads to overexpression of PI4P in HCV-infected cells [112,113]. Enteroviruses also co-opt PI4P as a host factor for replicating their genome. However, the kinase involved in enterovirus replication is PI4KIIIβ, and not PI4KIIIα [120]. PI4KIIIβ has also been reported to be involved in HCV replication in some studies [107,109,121], but not in others [103,105,110]. The origin of discrepancy between these studies is still unclear. The normal cellular function of PI4KIIIα is to produce PI4P as an intermediate for PI(4,5)P2 synthesis in the plasma membrane [122]. In contrast, the PI4P produced in HCV-infected cells does not appear to generate PI(4,5)P2 or other phosphoinositides. Therefore, not only the kinase but also its product are diverted from their original function by HCV.

In HCV infected cells, PI4P appears to function as a beacon for recruiting host factors at replication complexes. Recently, two PI4P-interacting lipid transfer proteins, OSBP and FAPP2, have been reported to be recruited to replication complexes in a PI4P-dependent manner and be required for HCV replication [123,124]. OSBP is a non-vesicular lipid transporter, which exchanges PI4P and cholesterol at ER-Golgi contact sites [125]. To perform this function, it interacts with VAP-A in the ER membrane, and to PI4P and Arf1 in the Golgi membrane. Interestingly, all these OSBP-interacting partners have been reported to be involved in HCV replication [121,126,127]. OSBP transfers cholesterol from the ER to the Golgi and uses an ER-Golgi gradient of PI4P produced by the Golgi-resident kinase PI4KIIIβ and the ER-resident PI4P phosphatase Sac1 to back-transfer PI4P from the Golgi to the ER [125]. By analogy, OSBP may function as a PI4P/cholesterol exchanger between ER and HCV replication complex apposed membranes and transfer cholesterol from the ER membrane to the PI4P-enriched membrane of the replication complex [124]. Much like NS5A and PI4KIIIα inhibitors, a small molecule inhibitor of OSBP inhibits HCV replication and generates morphologically aberrant NS5A-positive structures [124]. A similar PI4P-OSBP-cholesterol pathway controlled by PI4KIIIβ is also used by poliovirus [128,129]. In addition, OSBP could also regulate the assembly/secretion step of the HCV life cycle [130].

FAPP2 is a transporter of glucosylceramide [131]. It is structurally related to OSBP and was proposed to function in non-vesicular transport of glucosylceramide, a precursor of complex glycosphingolipids. Using inducible shRNA-mediated FAPP2 depletion and expression of mutant FAPP2 constructs, FAPP2 was recently shown to promote HCV replication in a PI4P-dependent manner by participating in the transport of glucosylceramide to HCV replication complexes, where it is converted in more complex glycosphingolipids, such as lactosylceramide and globotriaosylceramide [123]. These two glycosphingolipids were shown to be enriched in HCV replication complexes and their addition to FAPP2-depleted cells could rescue HCV replication [123]. These studies indicate that OSBP and FAPP2, two PI4P effectors required for HCV replication, provide cholesterol and sphingolipids to membranes of the replication complexes, and probably participate in the generation of a favorable lipid environment for the replicase.

### 4.3. Interplay between HCV Replication and Phospholipids and Fatty Acid Metabolism

In addition to the PI4P-cholesterol pathway, some studies also suggest a critical role of the metabolism of fatty acids and phospholipids during HCV replication. Not surprisingly, the generation of new membranes during HCV replication likely requires phospholipid synthesis. Accordingly, the expression of a number of genes involved in lipid metabolism is modulated during HCV infection [132,133,134]. This modulation of the lipid metabolism is probably controlled by SREBP [135,136]. Thus, fatty acid synthase is required for HCV replication and upregulated during HCV infection [134,137,138]. Other enzymes involved in fatty acid synthesis have also been reported to be involved in HCV replication [133,139,140,141]. This increase in fatty acid synthesis is likely used, at least in part, for making new membranes. Additionally, fatty acid synthesis may also be important for palmitoylation of viral proteins core and NS4B, which is required for their function [142,143]. In line with an essential role of the phospholipid metabolism, a modification of the phospholipids ratio correlates with replication inhibition [46]. Cytosolic phospholipase A2 gamma 4C (PLA2G4C), an enzyme that remodels phospholipids, and proline-serine-threonine phosphatase-interacting protein 2 (PSTPIP2), a host membrane-deforming protein, were shown to play a role in HCV replication [144,145]. Not only glycerophospholipids, but also host sphingolipid pathway was shown to be important for HCV replication [123,146,147,148].

Consistent with a role of fatty acid synthesis in promoting HCV replication, the exogenous addition of saturated and mono-unsaturated fatty acids has been shown to increase the replication of a genotype 1b replicon [133]. However poly-unsaturated fatty acids (PUFAs) are inhibitory [133]. This inhibition was shown to result from PUFA peroxidation, which could be blocked by vitamin E [149]. In addition, PUFA-enriched liposomes were proposed to exert an antiviral activity through the reduction of cellular cholesterol [150]. Recently, Yamane *et al.* found that HCV replication induces a sphingosine kinase-2-mediated lipid peroxidation of endogenous PUFAs, and confirmed that HCV replication is also sensitive to PUFA peroxidation [151]. These concomitant induction and sensitivity to lipid peroxidation constitute a feedback mechanism for keeping HCV replication at low levels, which may be an adaptation to chronicity. An interesting aspect of this study is the finding that the JFH1 strain, which replicates at high levels in cell culture, is insensitive to inhibition by lipid peroxidation. All other strains tested were sensitive and their replication efficiency could be greatly improved by vitamin E or sphingosine kinase-2 inhibitor SKI. This regulation by lipid peroxidation is proposed to participate in long-term persistence of the virus in infected patients [151].

### 4.4. Role of GBF1-Arf1-COP-I Pathway in HCV Replication

GBF1, a major regulator of membrane dynamics in the early secretory pathway, has recently emerged as a host factor involved in the replication of HCV [152] and several other positive RNA viruses of the *Picornaviridae* [153,154], *Coronaviridae* [155], and *Flaviviridae* [156] families. GBF1 is a brefeldin A (BFA)-sensitive guanine nucleotide exchange factor (GEF) of G-proteins of the Arf family. Arfs recruit and activate a number of effectors, which function in vesicular transport, phospholipid metabolism, non-vesicular lipid transport and actin cytoskeleton regulation [157,158]. The mechanism of action of GBF1 in viral infections is not yet fully understood. GBF1 inhibition by

BFA has a more profound effect at the beginning of the replication than at later time points for HCV [152], dengue virus [156] and mouse hepatitis virus [155]. However, the formation of membrane rearrangements is not inhibited by GBF1 inhibition [152,153,155], indicating that GBF1 is involved in the maturation or the activity of viral replication complexes, but not in their formation. During viral infections, GBF1 is generally assumed to function as an ArfGEF by activating Arf1, which in turn would recruit the COP-I coatomer, molecular machinery involved in intracellular transport, which has also been shown to be required for the replication of several positive RNA viruses [105,159,160]. Therefore, a GBF1-Arf1-COP-I pathway has been proposed to play a role in the replication of HCV [105,121,127,161]. This pathway mediates the retrograde transport from the cis-Golgi and the ERGIC to the ER [157], and is also implicated in the biogenesis of LD [162,163,164]. Accordingly, GBF1 could control the transport of a host factor (protein or lipid) essential for the viral replication. Indeed, it has been proposed to play a role in HCV replication by regulating the steady-state localization of the PI4P phosphatase Sac1 and consequently the levels of PI4P in HCV infected cells [165]. In addition, GBF1 may as well function in HCV replication by activating other cellular effectors. The activation of PI4KIIIβ, another Arf1 effector, by GBF1 has also been proposed to be involved in HCV replication [121]. In support of additional GBF1 functions, we recently isolated a series of BFA-resistant cell lines derived from Huh-7 cells, in which a partially active secretory pathway in the presence of BFA does not support HCV replication, suggesting a distinct mechanism of action of GBF1 in the protein secretory pathway and in HCV replication [161]. Other possibilities for GBF1 function during viral replication include mechanisms unrelated to Arf activation. For example, GBF1 function during poliovirus replication has been demonstrated not to depend on its catalytic Sec7 domain and therefore on Arf1 and COP-I activation [166]. This suggests that in addition to Arf activation GBF1 has unknown cellular functions that viruses could hijack.

## 5. HCV Assembly

HCV particle assembly assumes the spatial and temporal synchronization of the structural proteins and the replication complexes to result in the budding of an enveloped nucleocapsid. The involvement of the non-structural proteins in the assembly process constitutes a peculiarity of the *Flaviviridae* family [167]. For the ease of presentation, we will separate the assembly process into an early stage and a late stage, respectively. In the early stage, the viral modules involved in the process have to form and localize in the proximity of the assembly site, in order to assemble the RNA-containing nucleocapsid. In the late stage, the viral particle acquires an envelope, budding in the ER lumen, and matures within the secretory pathway overlapping with the VLDL secretion pathway.

### 5.1. Lipid Droplets and HCV Assembly Modules

Until now, the LD environment is considered the assembly site of HCV particle since all the viral factors involved in the process localize in the proximity of this organelle [168]. LDs are intracellular lipid deposits of cholesterol esters and triacylglycerides and inhibition of the synthesis of these lipids was recently shown to block HCV assembly [169]. LDs are surrounded by a phospholipid monolayer and they most probably originate from the ER by lipid accumulation between the ER phospholipid leaflets and budding into the cytosol or ER lumen (reviewed in [170]). These organelles travel on microtubules and their mobility and localization varies according to the LD associated proteome [171,172]. One of the most representative LD associated proteins is the adipose differentiation-related protein (ADRP), whose presence maintains the cytoplasmic distribution of LDs [173].

In the early stage, all the viral proteins form three different modules, which localize in proximity to the LDs: core, NS2 complex and the replication complex. Core protein comprises two domains: a N-terminal positively charged hydrophilic domain (D1) which interacts with the genomic RNA [174] and a hydrophobic domain (D2) which is the determinant of core attachment to ER cytosolic leaflet and further recruitment to LD [175]. D2 has two amphipatic helices, which are essential for core recruitment to LD and protein stability. NS2 is a transmembrane protein localized in the ER with a critical role in viral replication and assembly. Besides its function as a cysteine protease involved in NS2/NS3 cleavage, the role of NS2 in HCV assembly was quite elusive until recently, when several groups showed its interaction with E1, E2, p7 and NS3 to form a complex, which is recruited to virus-induced structures located in LD proximity [176,177].

### 5.2. HCV Core Recruitment to LD

HCV non-structural proteins NS3, NS4A, NS4B, NS5A and NS5B associate in the replication complex. As previously described, the viral RNA is replicated in special membranous structures called “the membranous web”. The replication complex is then recruited into proximity with LD in a core-dependent manner. Although all non-structural proteins were reported to be involved in HCV assembly, NS5A is the main assembly determinant in the replication complex. NS5A is a multifunctional protein composed of three domains: D1, D2 and D3 [178]. NS5A is involved in both replication and assembly through D1 [179] and D3 [180], respectively. NS5A may be modified post-translationally in D1 by phosphorylation resulting in a hyperphosphorylated form [181]. The hyperphosphorylation of NS5A is also dependent on the phosphorylation of a patch of serine residues in the C-terminus of D3 [182]. NS5A phosphorylation is deeply involved in HCV assembly, enabling core-NS5A interaction [183].

The current assembly picture assumes that the core protein attaches to LD through the D2 domain, dislocating the cellular ADRP in the process [175,184]. This determines a change in both size and subcellular distribution of LD, which concentrate in the perinuclear region as a consequence of core accumulation [184]. Intracellular localization of core with LD inversely correlates with the efficacy of viral assembly. Thus, in the context of mutations that inhibit viral assembly, the core protein accumulates around LD [185]. On the other hand, for viruses with efficient secretion, the core protein mainly shows an ER localization [186]. Interestingly, the motility of the core protein on LD was inversely correlated with the assembly efficacy, suggesting that the high motility of core would increase the odds of core reaching the LD where it would not take part in the assembly process [187]. These data suggest that core shuttles between the LD surface and the virion budding site, localized either at the ER or at the LD-ER interface. Recent life cell imaging studies have shown that core protein rapidly associates with LDs, and it is lately recruited into LD-independent small mobile structures. These mobile structures, likely corresponding to viral particles, move along the microtubules, following the secretion pathway along with apoE, but not apoB lipoprotein [185,188].

Core association with LDs is a crucial step for the recruitment of the other viral proteins to assembly sites [168]. This step was reported to be modulated by different host factors, mostly related to lipid metabolism and LD. Diacylglycerol acyltransferase 1 (DGAT1) performs the last step in triacylglycerol synthesis pathway together with DGAT2 and they are both involved in LD biogenesis. DGAT1 was shown to interact with core protein facilitating its recruitment to LD. While DGAT1 is involved in HCV assembly without affecting LD morphology, DGAT2 has no effect on HCV assembly [189]. Thus, DGAT1 is a host factor with a role in direct recruitment of core to LD.

Another class of host factors involved in HCV assembly would have an indirect role by interfering with LD biogenesis and subsequent core recruitment to LD. It is the case of the transcription factor IKK-alpha responsible for lipogenesis. While in the case of DGAT1 the LD morphology remains unchanged, IKK-alpha affects LD formation, LD-core interaction and HCV assembly [190].

Properties of lipid bilayers such as curvature and fluidity represent other factors that could influence both the core recruitment to LD and the budding process. Indeed, it was recently reported that HCV assembly needs the involvement of the cytosolic phospholipase A2 gamma 4A (PLA2G4A), an enzyme responsible for the specific hydrolysis of arachidonic acid from position 2 of phospholipids. PLA2G4A influences both the amount of core on LD, the core envelopment efficacy and the specific infectivity of the secreted particles [191].

### 5.3. HCV Replication Complex Recruitment to LD

The next major event in the early stage of HCV assembly is the recruitment of the replication complexes to LD, which depends on the presence of NS5A. If NS5A is expressed without other non-structural proteins, it localizes both in the ER and quite abundantly around LDs [192], while in the context of HCV subgenomic replicons, NS5A is located in puncta which are associated with the ER, but not with the LD [168]. In the presence of core protein, NS5A relocates extensively to the proximity of LD through a physical interaction between the hyperphosphorylated form of NS5A and the core protein [183,193]. Furthermore, NS5A hyperphosphorylation correlates with the assembly step [193], while the hypophosphorylated form of NS5A favors genomic replication [194]. Besides the core protein, it is possible that other host factors are involved in replication complex recruitment to LD. It was also recently reported that Rab18 interacts with NS5A facilitating recruitment of the viral protein to LD and HCV assembly [195]. Moreover, NS5A interaction with DGAT1, also influences its recruitment to the LD [196] making DGAT1 a central factor for viral assembly, responsible for both core and NS5A localization and interaction.

NS5A is also responsible for the recruitment to the LD surface of the GTPase Rab1 and the Rab1-GAP TBC1D20, which are responsible for both LD homeostasis and promotion of the HCV infectious cycle [192]. Moreover, the interaction of NS5A with apoE, identified as another crucial interplay for the viral assembly, also suggests a potential role for NS5A in the recruitment of this apolipoprotein to the assembly sites [197,198]. The indirect interaction of the negatively charged phospholipid binding protein annexin A2 with NS5A also seems to have an impact on HCV early assembly [199]. Thus, it appears that the recruitment of NS5A along with the replication complexes to LD represents a major step in the debut of HCV particle assembly and possibly the event that represents the transition from replication to assembly (reviewed in [200]).

### 5.4. HCV NS2 Complex

The third event characterizing early assembly implies the arrival of the envelope proteins at the assembly site. Different groups showed that E1 and E2 envelope glycoproteins are part of the NS2 complex, composed by NS2, p7 and NS3. Transmembrane domains of E2 and NS2 are key determinants in complex formation and NS2 subcellular localization [176,177,201,202]. Recently, the signal peptidase subunit 1 (SPCS1) was identified as a cellular factor involved in this complex formation. This host protein was reported to facilitate E2-NS2 interaction suggesting a cotranslational formation of the complex [203]. NS2 and envelope proteins, presumably as complexes, accumulate in NS5A positive dotted structures, which might represent replication complexes, and NS2-NS5A positive dots localize in close proximity of LD [176,177].

The late stage of HCV assembly debuts with core, the NS2 complex and the replication complexes in close proximity of LD. We may assume that virion budding probably derives from a combination of the pulling force resulting from lateral interactions of envelope proteins and the pushing force of the nascent nucleocapsid. Until now, our knowledge of HCV nucleocapsid envelopment is quite limited. However, as mentioned before, PLA2G4A might have a role in capsid envelopment and particle infectivity [191].

### 5.5. HCV Virion Budding and Secretion

Budding and secretion are other steps not completely elucidated. However, cellular proteins seem to play a central role during these processes. The Golgi-localized PI4P and the PI4P-binding protein GOLPH3 have been shown to have a role in HCV secretion. More precisely, GOLPH3 is involved in vesicle budding thanks to its interaction with the myosin MYO18A. Silencing of both these proteins leads to intracellular accumulation of viral particles and reduction of HCV secretion, suggesting a role in HCV budding [204]. The hepatocellular transcription factor Hepatocyte nuclear factor 4 (HNF4) is responsible for lipid metabolism and VLDL-mediated lipid transport. Downregulation of HNF4 or its downstream target phospholipase A2 GXIIB (PLA2GXIIB) also evidenced an impairment of HCV secretion [205]. In addition, other studies evidenced the colocalization of the Heat Shock Cognate Protein 70 (HSC70) with both E2 and core on the LD and its association with virions. Moreover, HSC70 downregulation determines an impairment of viral release, as well as a decrease of the volume of LDs [206].

These observations, as well as the low density of HCV viral particles and their association with apolipoproteins like apoB, apoE, apoC1 [20,22,31] led to the idea that HCV particle morphogenesis intersects the VLDL secretion pathway. VLDL formation begins with cotranslational association of apoB with the nascent lipoprotein formed by MTP mediated lipid accumulation between ER leaflets. Several reports showed that apoB and MTP are essential for HCV assembly [21,207]. Thus, one may imagine that the pushing force of lipid accumulation between ER leaflets during pre-VLDL biogenesis would lead to virion budding resulting in the formation of a LVP (Figure 3). This hybrid particle should be associated to apoB which was reported for both patient-derived and HCVcc virions [31]. On the other hand, later reports showed that while apoB is dispensable for HCV secretion, apoE and MTP are not [41]. Moreover, the mere presence of apoE can lead to the production of infectious particles in non-hepatic cells, suggesting the crucial role of this protein in HCV infectivity [208,209]. Interestingly, Hueging *et al.* showed that the role of apoE in HCV infectivity is in a post-envelopment step [209]. This would imply an apoE-HCV particle interaction in a post-budding step possibly by direct envelope-apoE interaction as it was recently reported [31,32]. A LVP as depicted in Figure 3 might be imagined as the interaction between intraluminal LD harboring apoE and an immature HCV particle. The nascent particle matures further in a post-ER step to get to the low density of an infectious particle [21]. HCV particle takes the secretory pathway and its secretion depends on classical host factors of the secretory pathway [188].

## 6. Conclusions

The effect of lipid metabolism on host-pathogen interactions has often been neglected in virology. The unique interaction between HCV and lipid metabolism offers the opportunity to deeply investigate the role of lipids in all the steps of the infectious cycle of a virus. The association of the viral particle with apolipoproteins and neutral lipids affects the way HCV interacts with the host cell at the entry step to generate a productive infection. Although the receptors and co-receptors of HCV are known, it is not clear if and how the lipidic part of the particle changes in the different stages of the entry process. Viral proteins induce profound changes in the intracellular membrane architecture and biochemical composition assuring the environment for viral replication. The picture of lipid dynamics and effector recruitment during the replication step is not complete and it awaits further development. Furthermore, viral assembly is associated with LD, it strongly depends on apoE and the particle seems to follow a similar maturation pathway as VLDL. However, the assembly step of infectious HCV particles leaves a series of aspects not yet addressed: the endogenous factors involved in the core envelopment step, the following sequence of events which lead to the formation of an infectious LVP and the molecular architecture of such a peculiar virion. Importantly, further studies of the interaction between HCV and lipid metabolism may also potentially help to better understand the role of some lipids in cell metabolism.
